# High Mobility Group Box 1 Induced Human Lung Myofibroblasts Differentiation and Enhanced Migration by Activation of MMP-9

**DOI:** 10.1371/journal.pone.0116393

**Published:** 2015-02-18

**Authors:** Chen-Chen Lee, Chien-Neng Wang, Yueh-Lun Lee, Yi-Ru Tsai, Jau-Jin Liu

**Affiliations:** 1 Department of Microbiology and Immunology, School of Medicine, China Medical University, Taichung, Taiwan; 2 Graduate Institute of Basic Medical Science, College of Medicine, China Medical University, Taichung, Taiwan; 3 Department of Microbiology and Immunology, School of Medicine, College of Medicine, Taipei Medical University, Taipei, Taiwan; Chang Gung University, TAIWAN

## Abstract

High mobility group box 1 (HMGB1) is a nuclear protein that involves the binding with DNA and influences chromatin regulation and transcription. HMGB1 is also a cytokine that can activate monocytes and neutrophils involved in inflammation. In this study, we investigated the role of HMGB1 on cellular activation using human fibroblast cell line WI-38. After treatment with 1, 10, and 100 ng/mL of HMGB1 for 24 h, we did not find obviously cytotoxicity and cellular proliferation of WI-38 cells by MTT and BrdU incorporation assay, respectively. However, we found that treatment with 10 and 100 ng/mL of HMGB1 induced the differentiation of lung fibroblasts into myofibroblasts and myofibroblasts showed higher migration ability through activation of matrix metalloproteinase (MMP)-9 activation. To delineate the mechanism underlying HMGB1-induced cellular migration, we examined HMGB1-induced mitogen activated protein kinases (MAPKs), including extracellular signal related kinase (ERK), c-Jun N-terminal kinase (JNK), and p38 mitogen activated protein kinase (p38) phosphorylation, as well as nuclear factor (NF)-κB nuclear translocation. Using specific inhibitors and shRNAs of protein kinases, we observed that repression of ERK, JNK, p38, and NF-κB all inhibited HMGB1-induced cellular differentiation, migration and MMP-9 activation in WI-38 cells. In addition, knocking down of RAGE but not TLR2 and TLR4 by shRNAs attenuated HMGB1-induced myofibroblast differentiation and migration. In conclusion, our study demonstrated that HMGB1 induced lung fibroblasts’ differentiation into myofibroblasts and enhanced cell migration through induction of MMP-9 activation and the RAGE-MAPK and NF-κB interaction signaling pathways. Targeting HMGB1 might be a potential therapeutic approach for alleviation of airway remodeling seen in chronic airway inflammatory diseases.

## Introduction

Airway remodeling is a prominent clinical feature in chronic asthma and chronic obstructive pulmonary disease (COPD) [1]. Airway remodeling causes lung tissue structure dysfunction, which includes damage of airway epithelium, goblet cells hyperplasia and mucus hypersecretion, subepithelial fibrosis and myofibroblast differentiation, and increase in smooth muscle mass [2,3]. These structural alterations contribute to the development of airflow limitation by increasing airway resistance. Thickening of the airway wall caused by fibrosis and inflammatory cell infiltration was found to be associated with the severity of asthma and COPD [4]. However, the cellular and molecular mechanisms underlying airway remodeling are still unclear.

Fibroblasts and myofibroblasts are key effector cells in the normal repair process of airway fibrosis. Fibroblasts and myofibroblasts are major sources of extracellular matrix (ECM) and facilitate homeostatic maintenance of ECM in the tissue. In asthma and COPD, dysregulation of fibroblast activation and differentiation into myofibroblasts leads to subepithelial fibrosis and remodeling of ECM in deeper airway. Previous studies have found that, in asthma patients, remodeling of ECM surrounding the airway smooth muscle cells might reduce airway elasticity, imposed load, and induction of excessive bronchoconstriction [5]. In the fibrotic process, fibroblasts are activated and recruited to the inflamed site by fibrogenic cytokines and growth factors such as tumor growth factor (TGF) β1 and platelet-derived growth factor (PDGF) [6].

High mobility group box 1 protein (HMGB1) was first reported as a nuclear protein that regulates gene expression and nucleosome stability [7]. During inflammatory reaction, HMGB1 is released into the extracellular region as a cytokine, stimulating the release of proinflammatory cytokines such as tumor necrosis factor (TNF) and interleukin (IL) 1α, IL-6, and IL-8 in monocytes [8], macrophages [9] and neutrophils [10]. HMGB1 also acts as a chemotactic factor that mediates the migration of monocytes and neutrophils. In addition, HMGB1 activates endothelial cells to upregulate adhesion molecules [11] and causes dendritic cells maturation [12]. HMGB1 binds to receptors, including advanced glycation products (RAGE), Toll-like receptor (TLR) 2, TLR4, and TLR9, to activate proinflammatory responses [13–15]. Downstream signaling mediated by HMGB1 interaction with these receptors include mitogen-activated protein kinases (MAPKs) and nuclear factor kappaB (NF-κB), and thereby facilitates cellular responses including cell migration [16] and release of pro-inflammatory cytokines.

Previous studies showed that HMGB1 is involved in the pathologic mechanism of pulmonary fibrosis-related diseases such as asthma, COPD [17], cystic fibrosis airway disease [18], and idiopathic pulmonary fibrosis [19]. In our previous study, we also found that localized blocking of HMGB1 expression in lung decreased collagen deposition and the production of remodeling factors TGFβ1 and vascular endothelial growth factor (VEGF)-1 [20]. Given the importance of HMGB1 with regard to pulmonary fibrosis, we hypothesized that HMGB1 may also directly influence lung fibroblast activation and it is still obscure.

To evaluate the potential role of HMGB1 on lung fibroblast activation, we cultured normal human lung fibroblast cell line WI-38 and investigated cell differentiation, proliferation, and migration after HMGB1 treatment. We found that HMGB1 induced the differentiation of lung fibroblasts to myofibroblasts, as well as enhanced cellular migration through MAPK and NF-κB signaling-dependent matrix metalloproteinase (MMP)-9 activation.

## Materials and Methods

### Cell culture

Human lung fibroblast cells, WI-38 cells, were purchased from Bioresource Collection and Research Center (Taiwan). WI-38 cells were cultured in Dulbecco's modified Eagle's medium (DMEM) supplemented with 10% heat-inactivated FBS purchased from Invitrogen (Carlsbad, CA, USA). Confluent cells were subcultured at a ratio of 1:3, and media were changed twice a week.

### Recombinant protein

Human HMGB1 recombinant proteins were purchased from R&D Systems (Minneapolis, MN, USA). Human TGF-β1 recombinant protein was purchased from Sigma Chemical Co (St. Louis, MO, USA).

### Cytotoxicity assay

The cytotoxicity was assessed by MTT (3-(4,5-Dimethylthiazol-2-yl)-2,5-diphenyltetrazolium bromide) assay following a previously described protocol [21]. WI-38 cells were cultured in 24-well-dishes and treated with various concentrations of HMGB1 for 24 h. At this point, added the 2 mg/ml 50 μL of 2 mg/mL MTT (Sigma) was added in each well and incubated for a further 3 h. The crystals formed in the cells were dissolved with DMSO, and the OD was measured at 570 nm.

### Knocking down gene expression by shRNA

Lentiviral expressed shRNAs were purchased from National RNAi core Facility Platform in Taiwan. Target sequences for different target genes sequences are shown in Table 1. Cells were infected with 3 multiplicity of infection (MOI) of lentiviral expressed shRNA for 48h and added 100ng/ml HMGB1 for further different time periods. Cells were collected for protein and mRNA analysis.

**Table 1:** shRNA targeting sequences.

Genes		Target sequence
**ERK**	si1	CAAAGTTCGAGTAGCTATCAA
	si2	GACATTATTCGAGCACCAACC
**JNK**	si1	CAGTAAGGACTTACGTTGAAA
	si2	GACTCAGAACACAACAAACTT
**p38**	si1	GTTACGTGTGGCAGTGAAGAA
	si2	CCATTTCAGTCCATCATTCAT
**NF-κB**	si1	GCCTTAATAGTAGGGTAAGTT
	si2	CCTGAGGCTATAACTCGCCTA
**TLR2**	si1	GCATCTGATAATGACAGAGTT
	si2	CCCATGTTACTAGTATTGAAA
**TLR4**	si1	GCCACCTCTCTACCTTAATAT
	si2	CCCTGCTGGATGGTAAATCAT
**RAGE**	si1	CGAGTCCGTGTCTACCAGATT
	si2	GCGGCTGGAATGGAAACTGAA

### Cell proliferation assay

WI-38 cells were cultured with different concentrations of HMGB1 for 16 h, and cells were pulsed with 5-Bromo-2-deoxyuridine (BrdU) for an additional 8 h. Cell proliferation was determined by BrdU incorporation assay according to the manufacturer’s instructions (Roche Diagnostics GmbH, Roche Applied Science, Germany) [22].

### Cell migration assay

Migration was determined using the wound healing assay [23]. Briefly, cells were seeded in 12-well plate and incubated to confluence. A cell-free wound area was then created by scratching the cells with a pipette tip. Cells were treated with different concentrations of HMGB1 and then allowed to migrate into the cell-free wound in serum-free DMEM for 18 h.

### Immunofluorescence staining of α-smooth muscle actin

Cells were cultured on cover-slips then treated with different concentrations of HMGB1 for 24 h or 16h. Cells were fixed with 4% paraformaldehyde and stained with anti-α-smooth muscle actin (α-SMA, Sigma) and DAPI for nucleus staining (Calbiochem, San Diego, CA, USA). Cells were then incubated with a secondary antibody, Alexa Fluor 488–conjugated goat anti-mouse IgG (Jackson ImmunoResearch Laboratories, Inc., Amish, PA, USA)[24]. α-SMA expression was observed using a fluorescence microscope (Zeiss, Carl Zeiss, Göttingen, Germany). The quantification of fluorescence intensity was performed by using Image J software.

### Real-time Quantitative PCR

RNA was converted into cDNA and then measured by quantitative real-time PCR using ABI PRISM 7900 Sequence Detector (Applied Biosystems, Foster City, CA, USA). The PCR product was amplified to obtain a statistically significant increase in target genes, which was determined using the threshold cycle (Ct) and normalized with GADPH [25]. The primer sequences are shown in Table 2.

**Table 2:** Primer sequences for real- time quantitative PCR.

Genes	Forward	Reverse
MMP-9	CGCGCTGGGCTTAGATCAT	GGTGCCGGATGCCATTC
ERK	TTAACACGGTGGACGAGTGG	GACACGACGTCAAAGGAGGT
JNK	GAAAACGCTGACTCAGAACAC	GCTGCACCTGTGCTAAAGGA
p38	TGGGATGCATAATGGCCGAG	AGAAACTCTTCTTCAAATCCCCA
NF-κB	AACAGCAGATGGCCCATACC	AATAGGCAAGG CAGGG GC
TLR2	TTGTGACCGCAATGGTATCTG	GCCCTGAGGGAATGGAGTTTA
TLR4	TGGTGTCCCAGCACTTCATC	GCCAGGTCTGAGCAATCTCATA
RAGE	GAGCCAGAAGGTGGAGCAGTA	GCAAGGGCACACCATCCT
GADPH	TGCACCACCAACTGCTTAGC	TCTTCTGGGTGGCAGTGATG

### Zymography

Cell medium was collected, and the total protein concentration was determined using the Bradford assay (Bio-Rad, Hercules, CA). Samples were loaded and separated by using 10% Tris-tricine gel with 0.1% gelatin as a substrate. After separation by electrophoresis, the gel was renatured and then incubated with development buffer at 37°C for 48 h. After development, the gel was stained with 0.5% Coomassie blue R-250 for 30 min and then destained appropriately [26]. The activity of MMP was quantified by using Image J software.

### Western blotting

The protein concentration of cell and tissue lysates was determined using a Bio-Rad protein assay kit (Bio-Rad, Richmond, CA, USA) according to the manufacturer’s instructions. Sodium dodecylsulfate polyacrylamide gel electrophoresis (SDS-PAGE) was performed on 10% polyacrylamide gels and then transferred onto polyvinylidene difluoride (PVDF) membranes (PerkinElmer Life Sciences, Boston, MA, USA) by the PantherTM Semidry Electroblotter (Owl Scientific, Portsmouth, NH, USA). The immunoblot was incubated overnight with blocking solution (5% skim milk) at 4°C, and then incubated with primary antibodies (Abcam, UK). After washing with Tris-Buffered Saline and Tween 20 (TBST), HRP-conjugated secondary antibodies (Amersham, Piscataway, NJ, USA, 1:5,000 dilution in TBST) were applied, and blots were developed on an enhanced chemiluminescence (ECL) detection system (PerkinElmer).

### Statistical analysis

All values are expressed as mean ± S.E.M. (n ≧ 3) and analyzed using one-way ANOVA followed by Newman-Keuls post-hoc comparisons. A *p* value < 0.05 was considered as statistically significant.

## Results

### HMGB1 induced myofibroblast differentiation and cell migration but not proliferation of human lung fibroblasts

**Fig 1:**
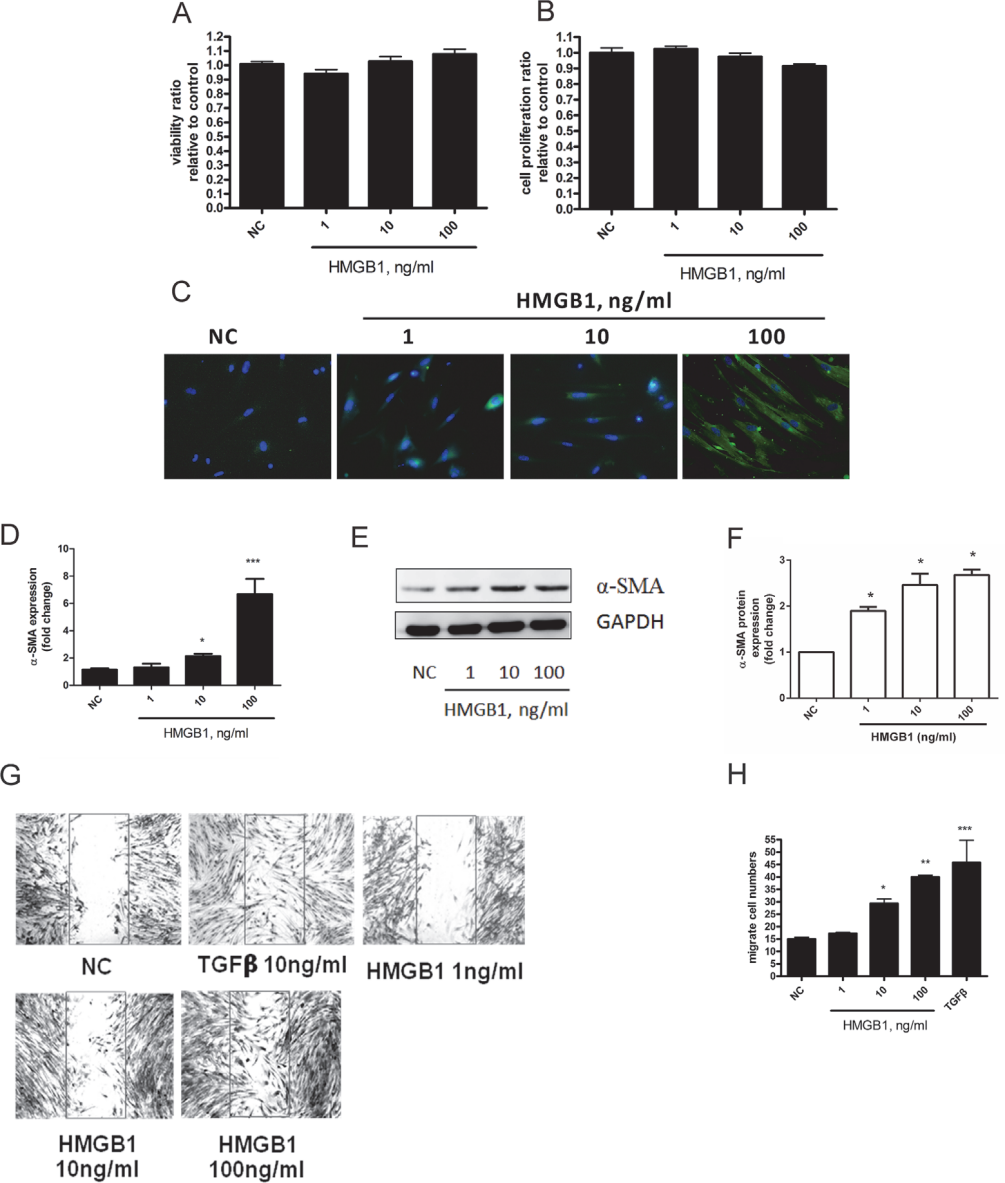
HMGB1 induced lung myofibroblast transition and migration in WI-38 cells. Cells were treated with 1, 10, and 100 ng/mL of HMGB1 for 24 h. (A) Cytotoxicity was detected by MTT. (B) Cell proliferation was detected by BrdU incorporation. (C) Myofibroblast transition was detected using α-smooth muscle actin (α-SMA) expression by immunofluorescence staining and quantification of α-SMA performed using ImageJ software (D). (E) Detection ofα-SMA expression by Western blotting and quantification of α-SMA performed using Image J software (F). (G) Cells were treated with 1, 10, and 100 ng/mL HMGB1 for 18 h. Cell migration was detected by *in vitro* scratch assay as described in Materials & Methods. (H) Quantification of cell migration was performed by counting the cell numbers in the rectangle in each sample for 4 fields. Data are expressed as mean ± S.E.M. (n ≥ 3) *p<0.05, **p<0.01, ***p<0.001 as compared with negative control (NC) group, in which the cells were treated with PBS.

A previous study showed that the concentration of HMGB1 in bronchial alveolar lavage fluid in healthy controls, asthma, and COPD patents was 3.7, 6.3, and 20 ng/mL, respectively [27]. Therefore, we used 1, 10, and 100 ng/mL of HMGB1 to investigate the effects of HMGB1 on fibroblast activation. First, we found that treatment with 1, 10, and 100 ng/mL of HMGB1 for 24 h did not induce cellular toxicity (Fig. 1A) in human lung fibroblast WI-38 cells. In chronic asthma and COPD airways, activated lung fibroblasts might initiate the transition to myofibroblasts, migration, and proliferation that contributes to airway fibrosis [28] [3]. Therefore, we elucidated the role of HMGB1 on lung myofibroblast differentiation, migration, and proliferation in WI-38 cells. After treatment, HMGB1 did not induce obvious cell proliferation in WI-38 cells; however, 10 and 100 ng/mL of HMGB1 induced obvious lung myofibroblast differentiation, which was detected by the staining of α-smooth muscle actin by immunocytochemistry (α-SMA, Fig. 1C and 1D) and western blotting (Fig. 1E and 1F). At 10 and 100 ng/mL HMGB1 treatment, HMGB1 induced obvious cellular migration in WI-38 cells. The higher level of lung myofibroblast differentiation in WI-38 cells indicated higher migration activity (Fig. 1G and 1H).

### HMGB1-induced WI-38 cell myofibroblast differentiation and migration through MAPK and NF-κB activation

**Fig 2:**
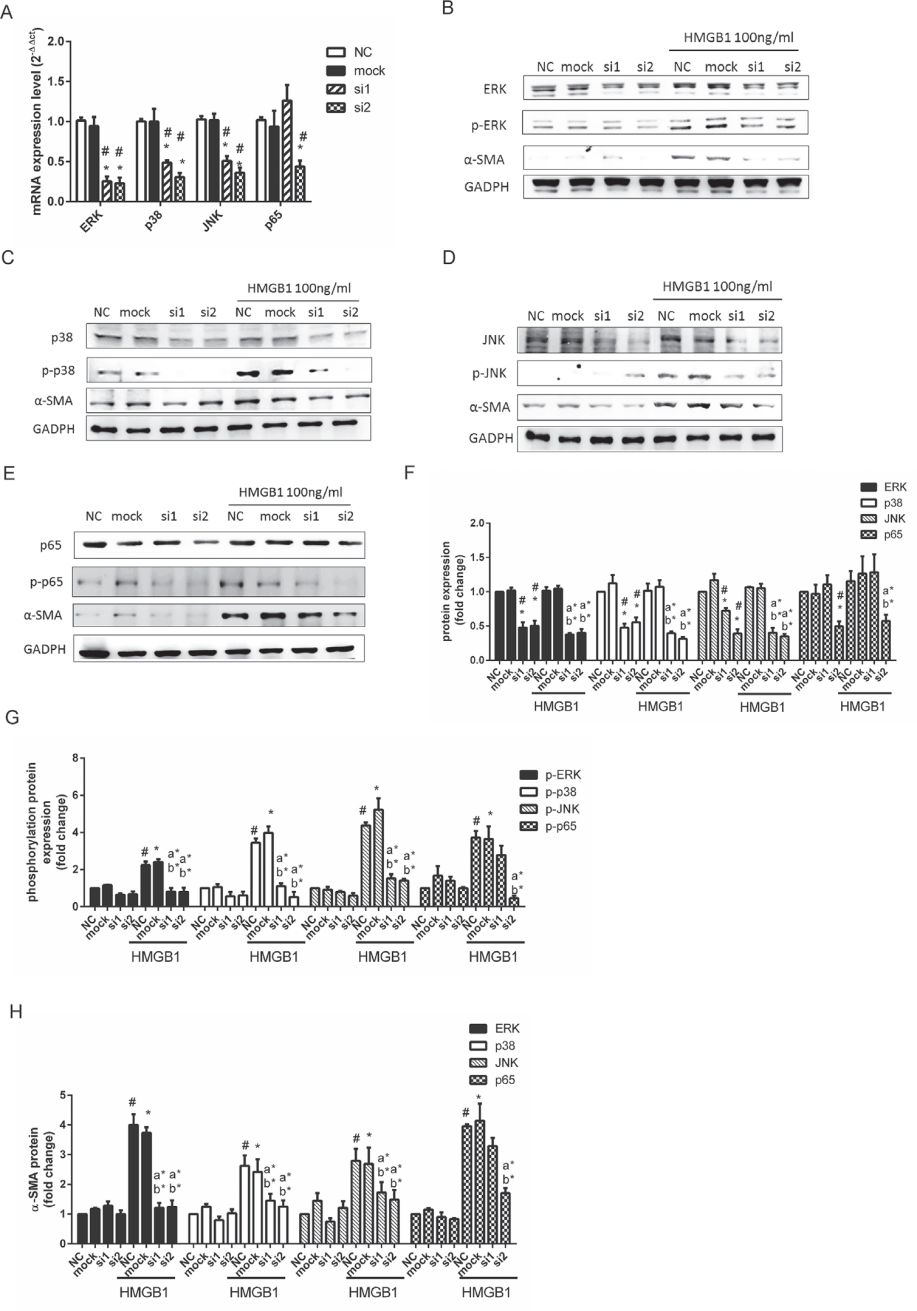
Knocking down of ERK, JNK, p38 and NF-κB dramatically inhibited HMGB1 inducedα-SMA expression. Cells were transfected with 3 MOI lentiviral based shRNA targeting ERK, JNK, p38, and p65 mRNA for 48h. Cells were collected for mRNA analysis by Real-time Quantitative PCR (A). Transformed cells were added with HMGB1 100 ng/mL for further 1h for phosphorylated proteins and 16 h for non-phosphorylated proteins. Cells were collected and analyzed protein expression by Western Blotting. (B) ERK, phosphorylated (p)-ERK, and α-SMA; (C) p38, p-p38, and α-SMA; (D) JNK, p-JNK, andα-SMA; (E) p65, p-p65, and α-SMA. Quantification of non-phosphosphorylated MAPK and p65 (F), phosphorylated MAPK and p65 (G), and α-SMA (H) performed using Image J software. Data are expressed as mean ± S.E.M. (n ≥ 3) #p<0.01 as compared with NC group. *p<0.05 as compared with mock group (lentiviral vector negative control). a*p<0.05 as compared with NC treated with HMGB1 100 ng/mL. b*p<0.05 as compared with mock group treated with HMGB1 100 ng/mL.

**Fig 3:**
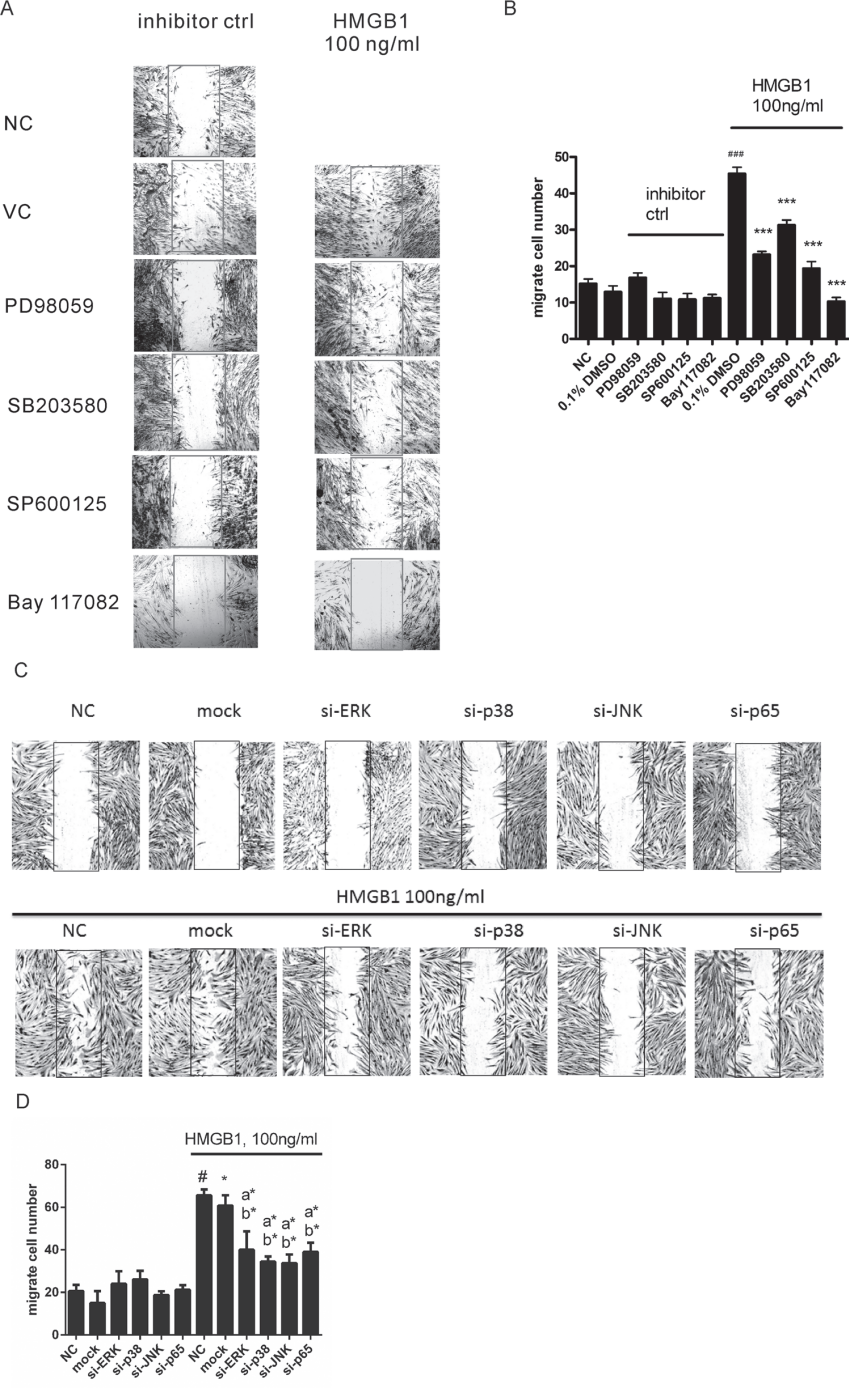
HMGB1 induced cell migration through activation of MAPK and NF-κB. Cells pretreated with 2 μM of different inhibitors (A) or transfected with lentiviral based shRNAs targeting ERK (si-ERK), p38 (si-p38), JNK (si-JNK), and p65 (si-p65) (C) were then treated with 100 ng/mL of HMGB1 for 18 h. Cell migration was detected by *in vitro* scratch assay. (B) (D) Quantification of cell migration was performed by counting the cell numbers in the rectangle in each sample for 4 fields. Data are expressed as mean ± S.E.M. (n ≥ 3) (B) #p<0.05 as compared with negative control (NC) group, in which the cells were treated with PBS. *p<0.05,**p<0.01, ***p<0.001 as compared to HMGB1 100 ng/mL group. (D) #p<0.01 as compared with NC group. *p<0.05 as compared with mock group (lentiviral vector negative control). a*p<0.05 as compared with NC group treated with HMGB1 100 ng/mL. b*p<0.05 as compared with mock group treated with HMGB1 100 ng/mL.

HMGB1 initiates important inflammatory reactions such as cytokine production, neutrophil migration, adhesion molecule expression, and proangiogenic gene expression through activation of MAPKs and NF-κB [29]. In addition, activation of MAPKs and NF-κB also involves in myofibroblast differentiation [30]. To clarify the mechanisms by which HMGB1 induced WI-38 cell differentiation and migration, we employed shRNAs of c-Jun N-terminal kinase (JNK), p38, extracellular signal-regulated kinase (ERK), and NF-κB as well as small molecular inhibitors of JNK inhibitor (SP600125), p38 inhibitors (SB203580), ERK inhibitor (PD98059), and NF-κB inhibitor (Bay117082) to investigate the signaling pathways (Figs. 2 and 3). WI-38 cells were transformed with lentiviral based shRNAs targeting ERK, p-38, JNK and p65. Two clones of ERK-si1 and -si2 (Fig. 2B), p38-si2 (Fig. 2C), JNK-si2 (Fig. 2D), and p65-si2 (Fig. 2E) showed reduction of an estimated 70% and 50% of target gene mRNA (Fig. 2A) and protein expression (Fig. 2F) as compared with mock group (lentiviral control). As compared with individual mock group (Fig. 2G), reduction of active form of ERK, p-38, JNK and p65 in ERK-si1, ERK-si2, p38-si2, JNK-si1, JNK-si2, and p65-si2 groups estimated 70% after HMGB1 treatment. After knockdown of ERK, p-38, JNK and p65, all shRNAs attenuated the HMGB1 induced α-SMA expression (Fig. 2H). Knockdown of ERK showed the highest inhibitory effects among different groups.

Next, we detected whether MAPKs and NF-κB regulated HMGB1-induced cell migration. Treatment with PD98059, SB203580, and SP600125 attenuated HMGB1-induced cell migration in WI-38 (Fig. 3A and 3B). Bay 117082 completely blocked HMGB1-induced cell migration in WI-38. To further confirmed HMGB1-induced cell migration mediated MAPK and NF-κB, cells were knocked down of ERK, p38, JNK, and p65 genes expression by shRNAs. We selected more effective clone of ERK, p38, JNK, and p65 shRNAs to investigate. As shown in Fig. 3C and 3D, ERK, p38, JNK, and p65 shRNAs displayed similar inhibition effects of cell migration as small molecular inhibitors; however, p65 shRNA did not completely block the HMGB1-induced cell migration in WI-38 (Fig. 3C and 3D).

**Fig 4:**
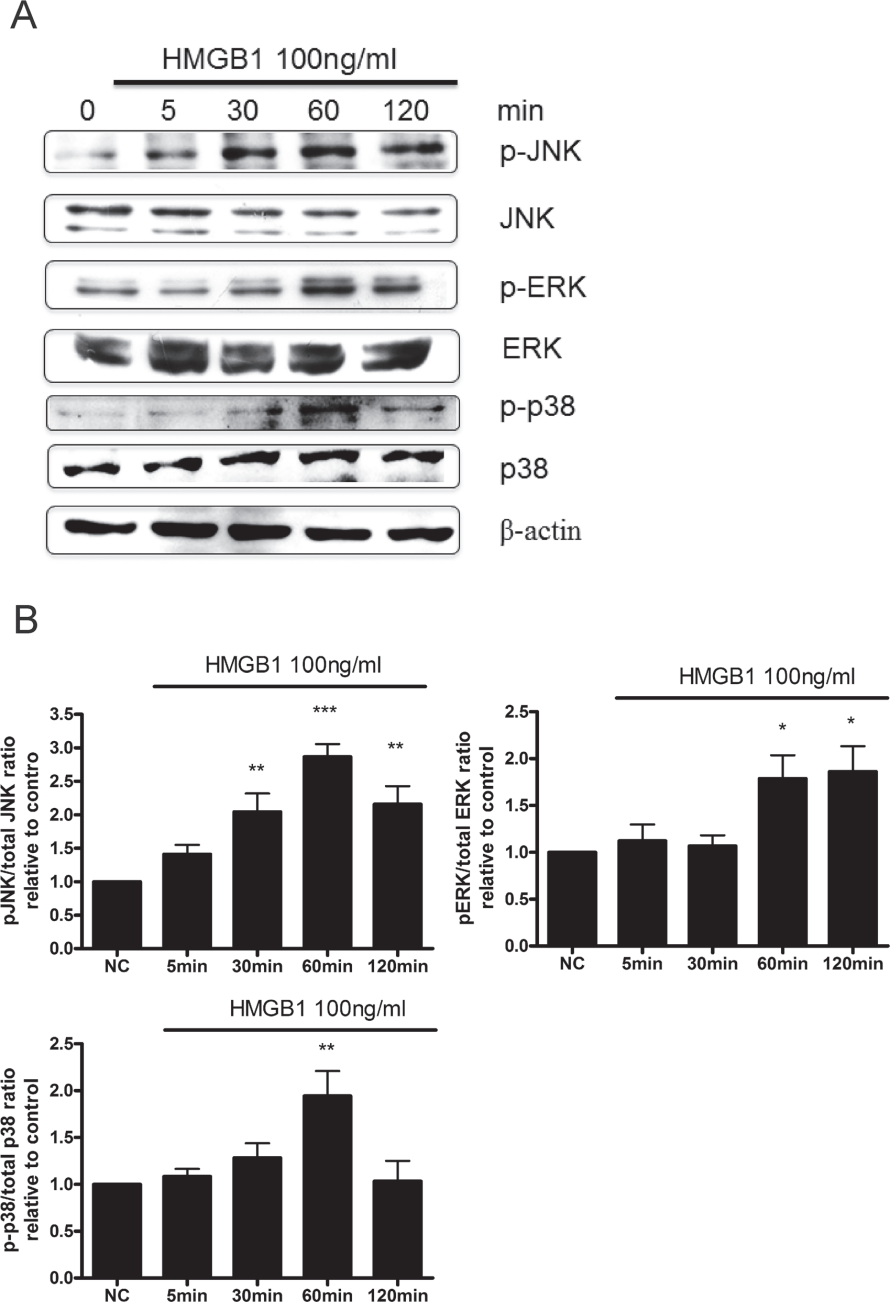
HMGB1 induced JNK, ERK, and p-38 phosphorylation in WI-38 cells. After the cells were treated with 100 ng/mL of HMGB1 for different time periods, protein was extracted, and the expression of phosphorylated and total JNK, ERK, p38 were analyzed by western blotting. β-actin was used as an internal control. (B). JNK, ERK, p38 phosphorylation were quantified by Image J software. Data are expressed as mean ± S.E.M. (n ≥ 3) *p<0.05,**p<0.01, ***p<0.001 as compared with negative control (NC) group, in which the cells were treated with PBS.

**Fig 5:**
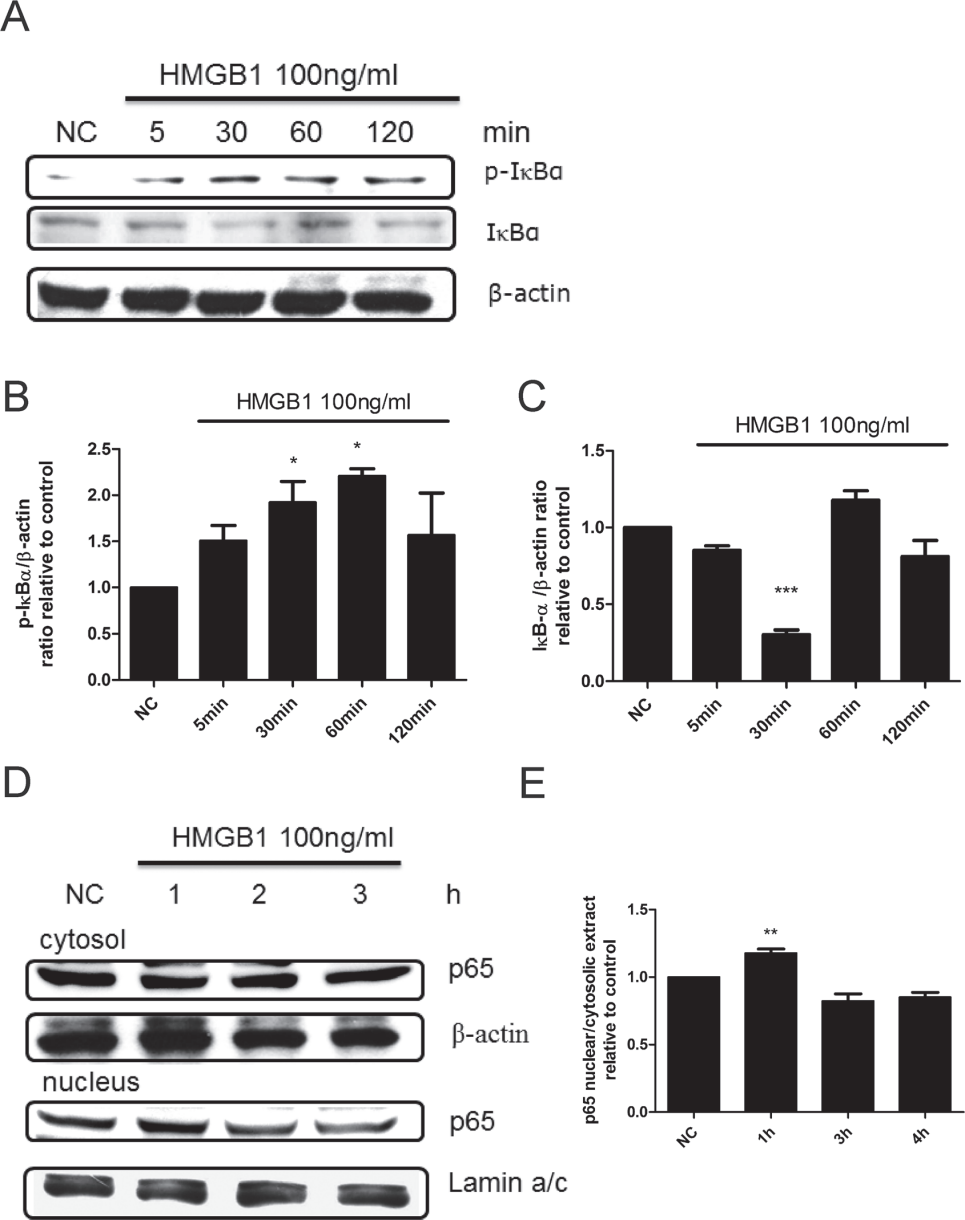
HMGB1-induced NF-κB activation in WI-38 cells. (A) After the cells were treated with 100 ng/mL of HMGB1 for different time periods, protein was extracted and phosphorylated, and total IκB was analyzed by western blotting. β-actin was used as an internal control. Phosphorylated (B) and total IκB (C) were quantified by Image J software. (D) Cells were treated with 100 ng/mL of HMGB1 for different time periods, and nuclear and cytosol proteins were isolated and p65 expression was analyzed. (E) Protein expression was quantified by Image J software. Data are expressed as mean ± S.E.M. (n ≥ 3) *p<0.05,**p<0.01, ***p<0.001 as compared with negative control (NC) group, in which the cells were treated with PBS.

Next, we directly examined the activation of MAPK and NF-κB after treatment of WI-38 cells with HMGB1. We found that 100 ng/mL of HMGB1 induced time-dependent JNK, p38, and ERK phosphorylation that reached a peak at 1 h (Fig. 4). We also found that HMGB1 induced NF-κB activation by phosphorylation of inhibitor of kappa B (IκB), degradation of IκB, and p65 translocation into nucleus (Fig. 5). Similar to MAPK activation, HMGB1-induced p65 translocation into nucleus reached a peak at 1 h (Fig. 5D and 5E).

### Involvement of MMP-9 but not MMP-2 activation in HMGB1-induced cellular migration

**Fig 6:**
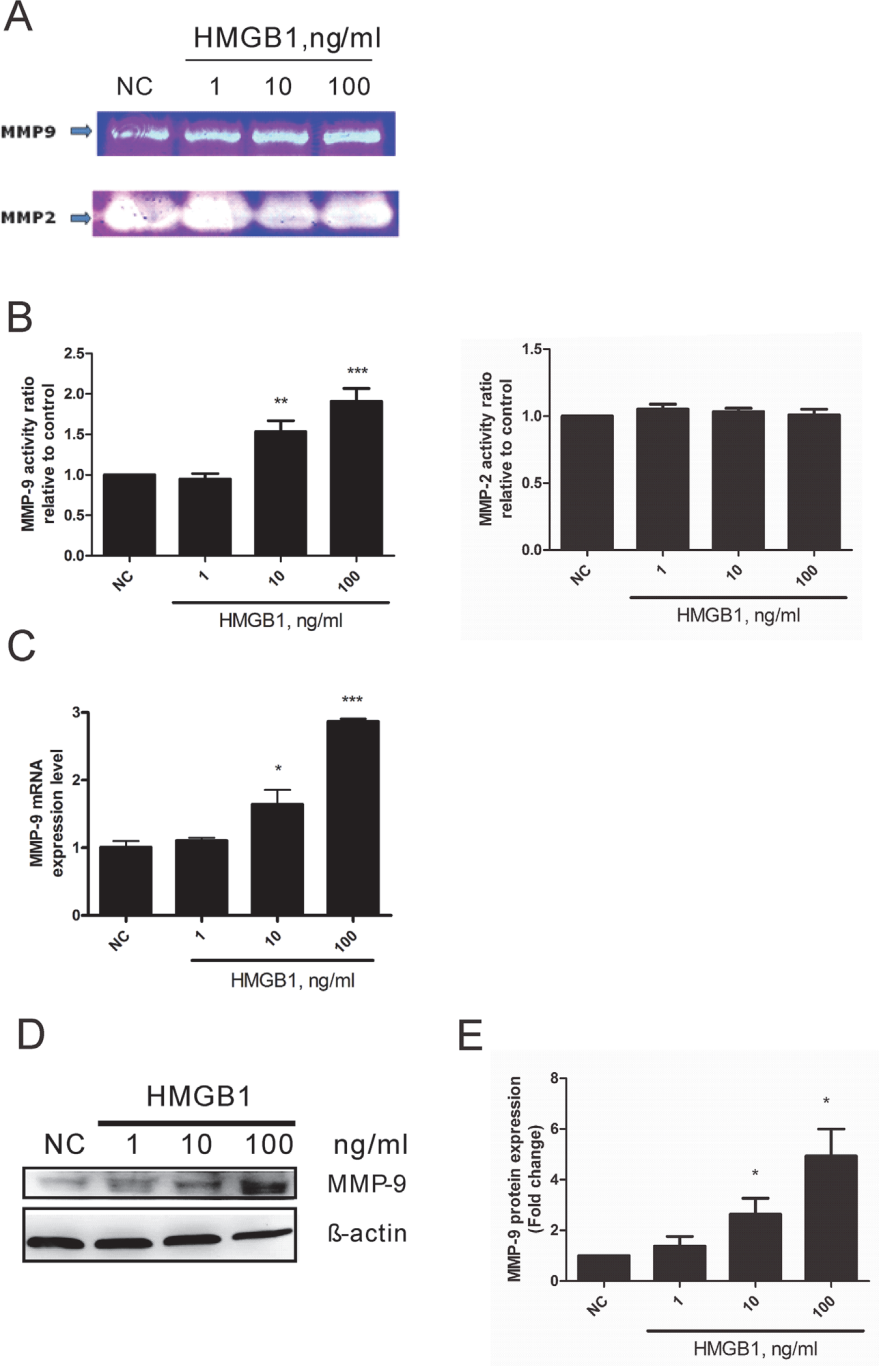
HMGB1 induced MMP-9 but not MMP-2 activation in WI-38 cells. Cells were treated with 1, 10, 100 ng/mL of HMGB1 for 18 h. (A). Cell culture medium was isolated for zymography. MMP-9 or -MMP-2 indicated active form of MMP-9 or MMP-2. (B). MMP-9 and MMP-2 expression was quantified by Image J software. (C). Cells were isolated for detection of MMP-9 mRNA expression by real-time PCR. (D). Cells were isolated for detection of MMP-9 protein expression by western blotting. (E). Protein expression was quantified by Image J software. Data are expressed as mean ± S.E.M. (n ≥ 3) *p<0.05, **p<0.01, ***p<0.001 as compared with negative control (NC) group, in which the cells were treated with PBS.

Myofibroblasts secrete MMPs to regulate extracellular matrix remodeling and induce cellular migration. Among MMPs, activation of MMP-2 and MMP-9 has been associated with pathogenesis of airway inflammation and remodeling [31]. Therefore, we wanted to know whether HMGB1-induced transition into lung myofibroblasts of WI-38 cells affects MMP-2 and -9 activities. As shown in Fig. 6A and 6B, 10 and 100 ng/mL of HMGB1 elevated MMP-9 activity but not that of MMP-2. To clarify whether HMGB1 regulated MMP-9 activity at the transcriptional and translational levels, we examined MMP-9 mRNA and protein expression in WI-38 cells and found that HMGB1 dose-dependently increased MMP-9 mRNA (Fig. 6C) and protein (Fig. 6D and 6E) expression.

### HMGB1-induced upregulation of MMP-9 activation through MAPK and NF-κB signaling pathways

**Fig 7:**
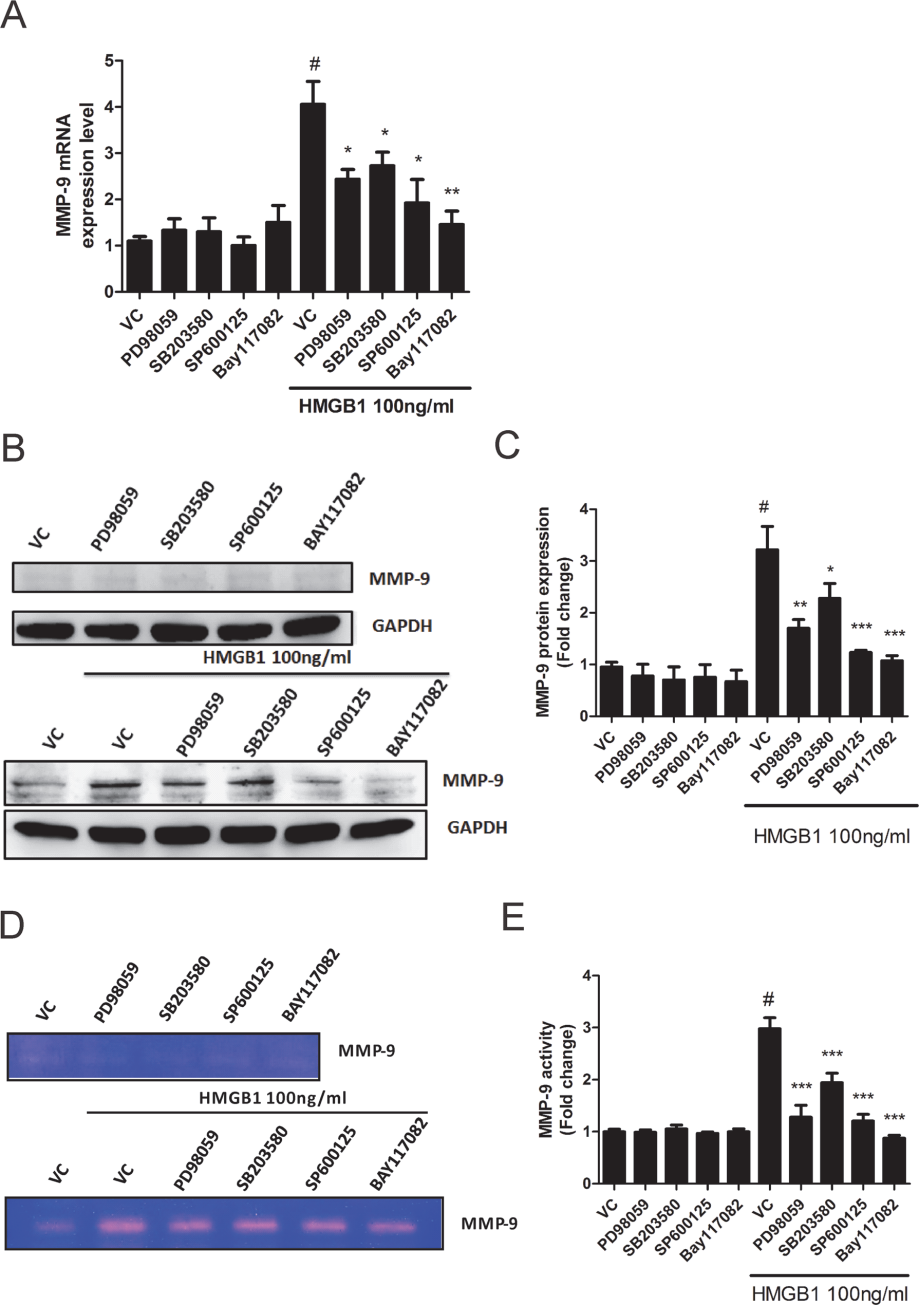
HMGB1 induced MMP-9 through activation of MAPK and NF-κB in WI-38 cells. Cells pretreated with 2 μM of different inhibitors were treated with 100 ng/mL of HMGB1 for 18 h. (A). Cells were isolated for detection of MMP-9 mRNA expression by real-time PCR. (B) Detection of MMP-9 protein expression was performed by western blotting. β-actin was used as an internal control. (C) Quantification of MMP-9 protein expression was done by using Image J software. (D) Cell culture medium was isolated for analysis of MMP-9 activity by zymography. MMP-9 indicated active form of MMP-9. (E) Quantification of MMP-9 activity was done by using Image J software. Data is expressed as mean ± S.E.M. (n ≥ 3) #*p*<0.05 as compared with negative control (NC), in which the cells were treated with PBS. *p<0.05, **p<0.01, ***p<0.001 as compared to HMGB1 100 ng/mL group.

Regulation of MMP-9 expression at the transcriptional level in lungs has been reported to occur through MAPK and NF-κB signaling cascades that are stimulated by growth factors, cytokines, and reactive oxygen species during chronic airway inflammatory diseases [32,33]. As shown in Fig. 3 and Fig. 4, HMGB1-induced cellular migration was decreased by inhibition of MAPK and NF-κB activation. These findings suggested a possible role of MAPK and NF-κB activation in HMGB1-induced MMP-9 activation. After treatment with PD98059, SB203580, and SP600125, HMGB1-induced MMP-9 activation, including mRNA expression (Fig. 7A), protein expression (Fig. 7B and 7C), and activity (Fig. 7D and 7E), were all attenuated in WI-38 cells. Treatment with NF-κB inhibitor-Bay 117082 completely blocked HMGB1-induced MMP-9 activation, including mRNA expression (Fig. 7A), protein expression (Fig. 7B and 7C), and activity (Fig. 7D and 7E) in WI-38.

### HMGB1 induced myofibroblast differentiation and cell migration through RAGE but not TLR2 and TLR4

**Fig 8:**
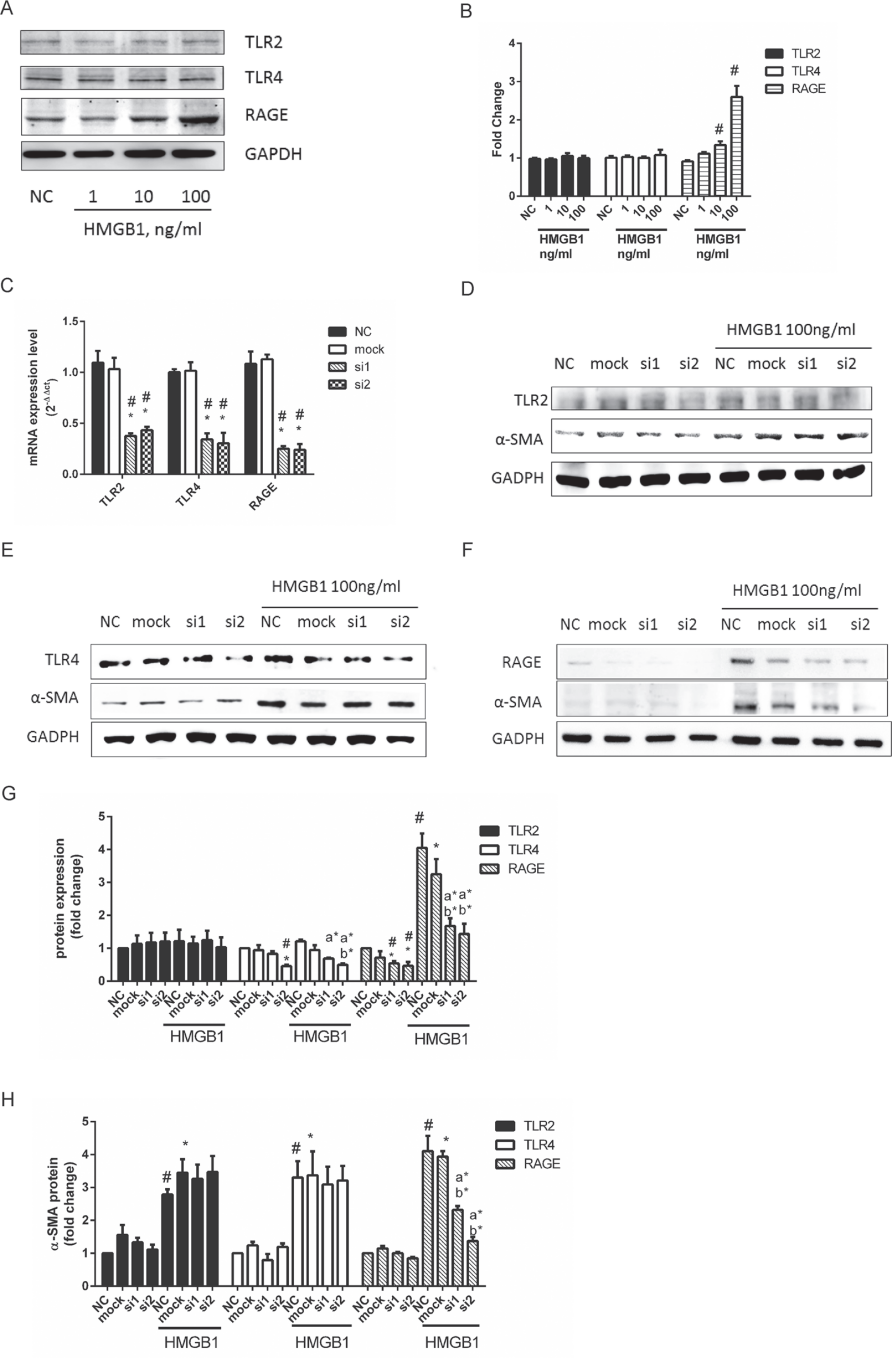
HMGB1-induced α-SMA expression mediated by RAGE. Cells were treated with 1, 10, 100 ng/mL HMGB1 for 18h. TLR2, TLR4, and RAGE were detected by immunofluorescence staining (A) and quantification of TLR2, TLR4, and RAGE performed using ImageJ software (B). Cells were transfected with 3 MOI lentiviral based shRNA targeting TLR2, TLR4 and RAGE mRNA for 48h. Cells were collected for mRNA analysis by Real-time Quantitative PCR (C). Transformed cells were added with HMGB1 100ng/ml for further 16 h. Cells were collected and analyzed protein expression by Western Blotting. (D) TLR2 and α-SMA; (E) TLR4 and α-SMA; (F) RAGE and α-SMA. Quantification of TLR2, TLR4, and RAGE (G) and α-SMA (H) performed using Image J software. Data are expressed as mean ± S.E.M. (n ≥ 3) #p<0.01 as compared with NC group. *p<0.05 as compared with mock group (lentiviral vector negative control). a*p<0.05 as compared with NC treated with HMGB1 100 ng/mL. b*p<0.05 as compared with mock group treated with HMGB1 100 ng/mL.

**Fig 9:**
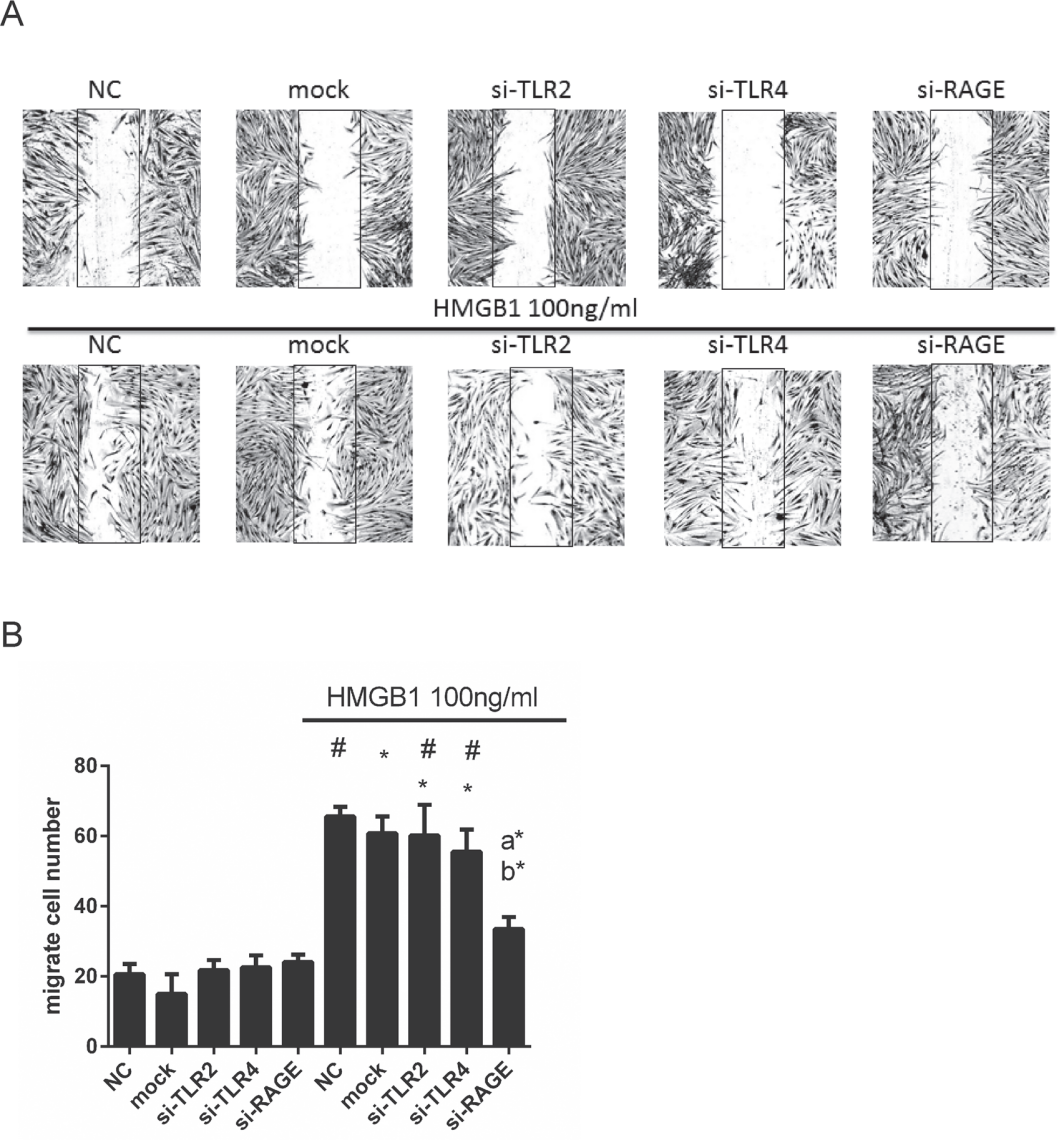
Knocking down of RAGE depressed HMGB1-induced migration ability. (A) Cells were transfected with 3 MOI lentiviral based shRNA targeting TLR2, TLR4 and RAGE mRNA for 48h. Cells were then treated with 100 ng/mL of HMGB1 for 18 h. Cell migration was detected by in vitro scratch assay. (B) Quantification of cell migration was performed by counting the cell numbers in the rectangle in each sample for 4 fields. Data are expressed as mean ± S.E.M. (n ≥ 3) #p<0.01 as compared with NC group. *p<0.05 as compared with mock group (lentiviral vector negative control). a*p<0.05 as compared with NC treated with HMGB1 100 ng/mL. b*p<0.05 as compared with mock group treated with HMGB1 100 ng/mL.

We next elucidated the role of the main cell membrane HMGB1 receptors, TLR2, TLR4, and RAGE, in HMGB1-induced myofibroblast differentiation and cell migration in WI-38. As shown in Fig. 8A and 8B, HMGB1 dose-dependently induce RAGE protein expression but not TLR2 and TLR4. WI-38 cells were transfected with lentiviral based TLR2, TLR4, RAGE shRNAs to knock down mRNA expression, respectively. Each clone of shRNAs of TLR2, TLR4, RAGE all showed obviously repression of an estimated 60% target mRNA expression as compared with mock group (Fig. 8C). As shown in the representative western blot, TLR4-si2 (Fig. 8E and 8G), RAGE-si1, and RAGE-si2 (Fig. 8F and 8G) except TLR2 shRNAs (Fig. 8D and 8G) after treatment of HMGB1 all knocked down of an estimated 60% target gene protein expression as compared with mock group. The reason why TLR2 shRNAs did not show inhibitory effect of target protein expression might be the level of TLR2 expression in WI-38 is too weak to detect. After TLR2, TLR4, and RAGE genes knockdown by shRNAs, HMGB1-induced α-SMA expression was only obviously inhibited in RAGE shRNAs infected cells (Fig. 8H). Similarly, TLR2- and TLR4- deficient cells failed to attenuate HMGB1-induced cell migration (Fig. 9). Only RAGE-deficient cells showed obviously inhibitory effects of HMGB1-induced cell migration.

## Discussion

### The pathological significance of HMGB1 on lung fibroblast differentiation to myofibroblast

Airway inflammation and remodeling are important clinical features of asthma and COPD. HMGB1 has been reported to play a crucial role in chronic airway inflammatory diseases through activation of eosinophils [34], neutrophils [10], and T helper (Th) 17 cells [20]. However, the role of HMGB1 in airway remodeling is still unclear. In this study, we demonstrated that HMGB1 induced the transition of human lung fibroblasts into myofibroblasts by inducing the higher expression of α-smooth muscle actin. This is the first study to directly demonstrate that HMGB1 can induce myofibroblast differentiation from lung fibroblasts.

Myofibroblasts are the key pathogenic cells in all fibrotic diseases. In tissue repair, myofibroblasts facilitate the contraction and closure of the wound through co-ordination with focal adhesions and ECM following apoptosis. However, in asthma and COPD, sustained airway inflammation causes dysregulation of myofibroblast differentiation and anti-apoptosis, which result in subepithelial fibrosis [35]. Our findings proved that HMGB1, which could be released from the damaged cells as well as phagocytes in damaged lung tissues [20], contributes to myofibroblast transition and migration that might cause subsequent subepithelial fibrosis.

The HMGB1-induced transition to myofibroblasts from lung fibroblasts was indicated by increases in migration but not proliferation. This is different from the classical fibrotic cytokine-TGF-β pathway that is well known for the induction of myofibroblast differentiation from fibroblasts, fibroblast and myofibroblast proliferation, as well as migration [35]. Therefore, HMGB1 probably does not have the characteristic features of a growth factor in lung myofibroblasts or fibroblasts. Besides, to determine whether myofibroblast transition increase cell migration activity, we treated cells with prostaglandin E2 (PGE2) which has been observed to inhibit fibroblast to myofibroblast differentiation [36]. We found PGE2 dramatically inhibited HMGB1 induced myofibroblast differentiation by inhibited α-SMA expression. Sequentially, HMGB1-induced cell migration ability attenuated (see S1 Fig.). Cell migration is a complex process that involves the ECM, MMPs, adhesion molecules, and chemotactic molecules. The importance of MMPs in cell migration is indicated by their binding to cell adhesion molecules, which facilitate proenzyme localization and activation and mediate cell motility by degradation of cell contacts with ECM [37]. In our study, HMGB1 probably induced lung myofibroblast migration through MMP-9 but not MMP-2. Previous studies found that the regulation of MMP activation is mediated at the transcriptional and posttranslational level [33]. Our study showed that HMGB1-treated lung fibroblasts upregulated MMP-9 activation both at the transcriptional and posttranslational levels, as reflected by the increases in mRNA and protein levels, and gelatinase activity. In addition, Lenga et al. showed that osteopontin most likely promotes cardiac fibroblast focal adhesion through increases in cellular HMGB1 expression [38]. However, the potential influence of HMGB1 on focal adhesion in order to promote cellular migration in lung fibroblasts still needs further investigation.

### Possible mechanisms of HMGB1-induced lung myofibroblast differentiation and migration

**Fig 10:**
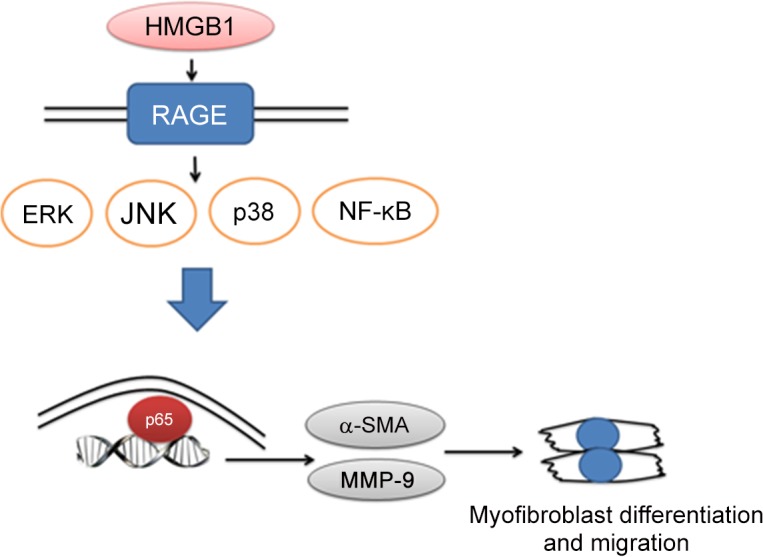
Schematic description of HMGB1-induced lung myofibroblast transition and enhanced migration in WI-38 cells.

The mechanisms underlying HMGB1-induced lung myofibroblast differentiation and migration have not been clarified. To clarify the signaling pathway, we examined MAPK and NF-κB activation, which are crucial regulators of multiple cellular functions, including migration, inflammatory cytokine production, survival, and differentiation [39]. We found that phosphorylation of ERK, JNK, and p38, and nuclear translocation of NF-κB, was seen in lung fibroblasts treated with HMGB1. Different MAPKs inhibitors and shRNAs were used to confirm which pathways contribute to HMGB1-induced lung fibroblast differentiation and migration. We found that HMGB1-induced lung myofibroblast transition and migration dramatically repressed after knocking down of ERK, JNK, p38 and p65. The present study reveals that activation of both MAPK and NF-κB crucially contributes HMGB1-induced myofibroblast transition and migration activity. We adopt small molecular inhibitors and shRNAs to inhibit MAPK and NF-κB activation. Results indicated the inhibitory effects between two different characteristics inhibitors have similar potency except NF-κB inhibitor. HMGB1-induced lung myofibroblast migration was completely blocked by NF-κB inhibitor-Bay117082 but not p-65 shRNA. As Bay117082 is an IκB–α phosphorylation inhibitor which will reduce both p50 and p65 nuclear translocation; therefore, the level with inhibition of downstream target genes is better than p65 shRNA which blocks p65 only. However, the exact mechanism need further investigate. HMGB1-activated MMP-9 gene expression and activity also showed a similar pattern, as observed in the lung myofibroblast migration after treatment with MAPK and NF-κB inhibitors. Therefore, HMGB1 causes activation of MAPKs (p38, JNK, and ERK) and NF-κB, and subsequent activation of MMP-9, which induces lung myofibroblast migration (Fig. 10).

HMGB1 membrane receptors- TLR2, TLR4, and RAGE are main receptors associated with HMGB1 induced inflammatory response. Our study reveals that HMGB1 induced myofibroblast differentiation and increased migration ability mediated through RAGE but not TLR2 and TLR4. Besides, HMGB1 induced RAGE protein expression but not TLR2 and TLR4 in WI-38. It is interesting because lots of studies has confirmed that RAGE mediates function of cell mobility of HMGB1 but less reports HMGB1-RAGE interaction is important for myofibroblast differentiation. HMGB1-TLR2 and TLR4 signaling mainly regulates cytokines production [40] and our study shows that TLR2 and TLR4 didn’t play a key role in myofibroblast differentiation and migration induced by HMGB1.

### Conclusion

The present study demonstrated that HMGB1 causes the differentiation of lung fibroblasts into myofibroblasts, which enhances cellular migration through activation of MMP-9 and the RAGE-MAPK and NF-κB interaction signaling pathways. This might emerge as an important therapeutic strategy for airway remodeling in chronic airway inflammatory diseases, including asthma and COPD, because progressive loss of lung function by airway remodeling still cannot halted by current treatment regimens. As lung myofibroblasts play a crucial role in subepithelial fibrosis and cytokine production during airway inflammation, targeting HMGB1 might have significant therapeutic potential for alleviation of the progressive loss of lung function seen in chronic airway inflammatory diseases.

## Supporting Information

S1 Fig PGE2 attenuated HMGB1 induced myofibroblast transition and cells migration. Cells were pretreated with 500nM PGE2 for 24h and then stimulated with HMGB1 100ng/ml for 16h for α-SMA protein analysis by western blotting (A) and quantification of α-SMA performed using Image J software (B). (C) After pretreated with 500nM PGE2 for 24h, cells were stimulated with HMGB1 100ng/ml for 18h for cell migration by in vitro scratch assay. (D) Quantification of cell migration was performed by counting the cell numbers in the rectangle in each sample for 4 fields. Data are expressed as mean ± S.E.M. (n ≥ 3) (B) #p<0.05 as compared with negative control (NC) group, in which the cells were treated with PBS. *p<0.05 as compared to HMGB1 100 ng/mL group.
(DOCX)
