# Supplementary material for: High Mobility Group Box 1 Induced Human Lung Myofibroblasts Differentiation and Enhanced Migration by Activation of MMP-9
Source: PLoS One. 2015 Feb 18;10(2):e0116393. doi: 10.1371/journal.pone.0116393 (PMC4332862; doi:10.1371/journal.pone.0116393)
Supplement: S1 Fig — Cells were pretreated with 500nM PGE2 for 24h and then stimulated with HMGB1 100ng/ml for 16h for α-SMA protein analysis by western blotting (A) and quantification of α-SMA performed using Image J software (B). (C) After pretreated with 500nM PGE2 for 24h, cells were stimulated with HMGB1 100ng/ml for 18h for cell migration by in vitro scratch assay. (D) Quantification of cell migration was performed by counting the cell numbers in the rectangle in each sample for 4 fields. Data are expressed as mean ± S.E.M. (n ≥ 3) (B) #p<0.05 as compared with negative control (NC) group, in which the cells were treated with PBS. *p<0.05 as compared to HMGB1 100 ng/mL group. (DOCX) [file pone.0116393.s001.docx]

Supporting information
